# Supplementary material for: Collaborative intelligence in AI: Evaluating the performance of a council of AIs on the USMLE
Source: PLOS Digit Health. 2025 Oct 9;4(10):e0000787. doi: 10.1371/journal.pdig.0000787 (PMC12510544; doi:10.1371/journal.pdig.0000787)
Supplement: S2 File — (ZIP) [file pdig.0000787.s002.zip › S2 Data Raw data files containing unprocessed question inputs and LLM outputs including transcripts of deliberations/STEP3_Question-60-1_Type-MC-NJ.docx]

**Facilitator** **[Round 1]:** A 19-year-old woman is referred to the office by her counselor for evaluation of possible depression. The counselor describes the patient as polite, conscientious, cooperative, open to therapy, and always present and on time for her weekly sessions. The patient's relationship with her high school boyfriend ended 6 months ago. She had dated him through high school, and he had ""looked out for her as a friend"" since the 6th grade. She says, ""I feel helpless and don't know what to do without him."" She admits that she often cannot make decisions, such as whether to go to college. She says, ""I always mess things up."" She is still sad and often tearful about her ex-boyfriend, but she has not had alterations in her sleep habits, appetite, weight, or energy level. She has not had any thoughts about death. Medical history is unremarkable and she takes no medications. BMI is 24 kg/m². Vital signs are normal and physical examination discloses no abnormalities. Which of the following is the most appropriate initial response to this patient's self-deprecating description? (A) ""College is full of many great once-in-a-lifetime opportunities. You should go."" (B) ""Everyone makes mistakes. In your case, though, that doesn't mean you always mess things up."" (C) ""If you find yourself needing some guidance, call me."" (D) ""You're overreacting. You just need to give it a little more time.""

**AI_B:** (1) RESTATING THE QUESTION: A 19-year-old woman presents with feelings of helplessness, sadness, and self-criticism following the end of a long-term relationship and is having difficulty making major life decisions such as going to college. Despite these feelings, she does not exhibit alterations in sleep, appetite, weight, or energy levels and denies thoughts of death. Her medical history is non-significant, she isn't on medications, her BMI is within normal, vitals signs are stable, and the physical exam is unremarkable. Our task is to determine the best initial response to her self-disparaging remarks. (2) PLANNING: Assumptions made could be that she is experiencing a depressive disorder or she is simply dealing with a difficult life event. It's also possible that her self-deprecation is a manifestation of low self-esteem or self-confidence. An alternative assumption might be that she may be experiencing a form of adjustment disorder due to the end of her long-term relationship. Clinically, it would be vital to rule out a major depressive disorder given her symptoms. (3) CLINICAL REASONING: Given the patient's age, the timing and nature of her symptoms (relationship end), and the absence of classic depressive symptoms such as altered sleep or appetite, a diagnosis of major depressive disorder seems slightly less likely although cannot be completely ruled out. Instead, her symptoms could be indicative of an adjustment disorder. Adjustment disorder refers to the development of significant emotional or behavioral symptoms in response to a stressor. In this case, the breakup acts as the stressor. Frequently, these symptoms can manifest as sadness, hopelessness, or difficulty making decisions. (4) CONCLUDING: Given the understanding above, an appropriate response would ideally acknowledge her feelings and provide a sense of support while not making any unequivocal statements about her future actions or decisions. It would help to show empathy, validate her feelings and emotions, and encourage her to converse about her concerns. (5) RESPONSE: Based on the above considerations, a suitable initial response would likely be option B: ""Everyone makes mistakes. In your case, though, that doesn't mean you always mess things up."" This would acknowledge her feelings, reassure her, and challenge her negative self-belief without passing sweeping judgements or decrying her worries.

**AI_C:** (1) RESTATING THE QUESTION: The given scenario introduces a 19-year-old woman who is referred to a health professional for possible depression. She recently experienced a breakup with her long-term boyfriend which has left her feeling helpless and uncertain, particularly about significant decisions like pursuing a college education. She consistently expresses negative views about herself, but doesn't show the classical signs of clinical depression such as sleep disturbances, altered eating habits, weight changes, low energy, or suicidal thoughts. Her overall health appears normal, including BMI and vital signs. The question asks for the most suitable initial feedback to her negative self-perception from the options given. (2) PLANNING: The assumptions here could be 1) the woman is depressed due to her breakup; 2) her self-deprecation is disastrous; 3) she cannot recover without professional help. We can challenge these assumptions by considering that she may be going through a typical grieving process after a significant life change, and that she might benefit more from supportive feedback and resilience-building than conventional psychiatric treatment. Clinically, it makes sense to prioritize the assumption that she requires thoughtful, respectful, empathetic response to her self-deprecation over an immediate move to medication or suggestion of pathological depression. (3) CLINICAL REASONING: Her situation seems more reflective of an adjustment disorder, a response to distressing life events, than clinical depression - she isn't showing key depressive symptoms and her distress arose after a clear precipitating event. Therefore, it's more beneficial to validate her feelings and let her know she could always ask for help, instead of pushing forward with the idea of depression. Challenging negative self-perception is paramount in helping build her resilience and self-esteem. A direct push to something like attending college might not be ideal as decision-making is part of what she's struggling with. (4) CONCLUDING: Given this clinical understanding and the choices available, we should focus on a response that 1) validates her feelings, 2) supports her in dealing with her self-deprecating tendency, and 3) encourages her autonomy and decision-making ability. (5) RESPONSE: Considering these points, the most appropriate response would be (B) "Everyone makes mistakes. In your case, though, that doesn't mean you always mess things up." This statement acknowledges her feelings, gently challenges her negative self-image, and does not rush her into making significant life decisions.

**AI_E:** RESTATING THE QUESTION: The problem at hand is discovering the most apt initial reply to a young woman's self-deprecating statements. She was sent to a professional by her counselor for potential depression. She expresses feelings of helplessness, trouble with decision-making, and sadness. Despite these symptoms, she has not had significant changes in her sleep, appetite, weight, or energy, nor has she thought about death. PLANNING: The primary assumption is that the described symptoms may indicate depression. Alternate assumptions could be that the patient is experiencing normal grief after a breakup or that she has a dependent personality disorder due to her difficulty making decisions and feeling of helplessness without a protective figure. Given her normal sleep, appetite, weight, energy level, and absence of thoughts about death, it can be hypothesized that she may not meet all the diagnostic criteria for depression. CLINICAL REASONING: Depression is a heterogeneous condition with a variety of symptoms. The patient’s lack of altered sleep, appetite, weight, energy level, and death thoughts does not completely rule out the diagnosis, but makes it less probable. Grief, while a normal reaction to loss, typically decreases in intensity over time, and it has been six months since the end of her relationship; so, it's probably not the sole explanation. Dependent personality disorder, on the other hand, may describe this patient’s dilemma well, as this disorder is characterized by a pervasive, excessive need to be taken care of, leading to submissive and clinging behavior, fears of separation, and difficulty making decisions. CONCLUDING: From the information given, it seems plausible that the young woman might be struggling more with a dependent personality disorder than with depression or prolonged grief. Thus, the response should be a supportive one that acknowledges her feelings and reinforces her capacity to cope and make decisions, rather than discounting her experiences or encouraging dependency. RESPONSE: Based on the above reasoning, the most appropriate response seems to be: (B) "Everyone makes mistakes. In your case, though, that doesn't mean you always mess things up." This gives the patient an opportunity to view her circumstances from a different perspective and reinforces her ability to cope and make decisions.

**AI_D:** (1) RESTATING THE QUESTION: The situation involves a 19-year-old woman who is delivered to the clinic for a probable depression analysis. Although the young woman is currently experiencing emotional distress, the clinical parameters of major depressive disorder - alterations in sleep, appetite or weight, energy levels or suicidal thoughts - aren't observed in her symptoms. The task at hand is determining how to respond appropriately to her negative self-evaluation. (2) PLANNING: The assumptions behind the presented choices largely concern the empathetic versus directive approaches in therapy. Should the clinician endorse her potential choices (Choice A), provide her with reassurance (Choice B/C), or directly challenge her perspective (Choice D)? The therapeutic alliance is unaffected by choice A because it assumes a decision has already been made. It's likely, however, that choice B, which provides reassurance, could help diminish her self-deprecating tendencies and foster her self-esteem. Choice C, providing a personal lifeline of sorts, might help instill a sense of safety and trust in the therapeutic relationship but won't specifically address her negative self-evaluation. Lastly, choice D might challenge her perception but risk rupture of the therapeutic alliance, as it may dismiss her emotional experience as an overreaction. (3) CLINICAL REASONING: It's important to understand how the style of psychotherapy can influence its effectiveness. An empathetic and validating response would be more connected to patient-centered therapy, which allows patients to explore their own solutions and build resilience. This is crucial in creating a safe therapeutic environment and building a strong therapeutic alliance which is an essential part of successful therapy. In contrast, a less considerate response risks antagonizing the patient and pressuring her to suppress her feelings which can lead to a more complicated recovery process. (4) CONCLUDING: A response that acknowledges the patient's feelings and struggles while also encouraging her to see her abilities positively will generally foster healing and resilience. Given this, the response that seems most advantageous would validate her feelings and challenge her negative self-talk by reassuring her that it's normal to make mistakes, and that it doesn't mean she always messes things up. (5) RESPONSE: Based on the above reasoning, the most appropriate initial response to this patient's self-deprecating portrayal would be (B) ""Everyone makes mistakes. In your case, though, that doesn't mean you always mess things up."" This response validates her feelings and challenges her belief system without being dismissive or directive, potentially supporting her self-esteem and therapeutic alliance.

**AI_A:** RESTATING THE QUESTION: The inquiry provided concerns the best initial response to supply a young woman who is potentially dealing with depression, following the end of a significant relationship. Her symptoms include feelings of helplessness and a belief that she always makes mistakes. Despite the pain caused by the breakup, she does not show alterations in appetite, sleep habits, weight, energy level, or suicidal ideation. Her BMI and vital signs are normal, with no abnormalities detected in her physical examination. PLANNING: The key assumptions we could make based on the details provided are: she may be suffering from depression or an adjustment disorder due to a significant life event (the breakup). However, considering her lack of alterations in sleep, appetite, weight, and energy level, it would suggest that she may not clinically qualify for a diagnosis of depression. The absence of suicidal ideation is also significant here. Alternatively, it might be that her self-deprecation is symptomatic of low self-esteem, resulting from the end of a supportive relationship. CLINICAL REASONING: While depression is a valid assumption under these circumstances, it might not be the only explanation for her symptoms. It is crucial to remember that depression should not be diagnosed unless specific diagnostic criteria are met. According to DSM-5, these usually require the presence of a minimum of five symptoms (persistent feelings of sadness, loss of interest, changes in appetite or weight, sleep issues, decreased energy, feelings of worthlessness, reduced concentration, psychomotor agitation or retardation, and recurrent thoughts of death) for a duration of at least two weeks, causing significant distress or impairment in social or occupational functioning. And since the young woman only reports persistent sadness and feelings of worthlessness, she may not meet the clinical criteria for a diagnosis of depression. Therefore, the symptoms she presents may be more indicative of an adjustment disorder or low self-esteem related to the loss of her relationship. CONCLUDING: When dealing with a patient who expresses feelings of worthlessness and uncertainty, it's essential to respond in a way that validates her emotions and feelings, creates a supportive environment, and promotes self-worth autonomously, not based primarily on external relationships or achievements. It is also important to provide resources for help if needed. RESPONSE: Given these insights, the most appropriate initial response would be to reassure the woman that everyone makes mistakes and that everyone's case is unique. Therefore, option (B) ""Everyone makes mistakes. In your case, though, that doesn't mean you always mess things up,"" would be the most appropriate response. This approach subtly reassures her that mistakes are part of being human and encourages her to disassociate her worth from occasional errors. The response also challenges her distorted self-perception and promotes positive self-regard, both of which are important therapeutic interventions.

**Facilitator** **[Round 2]:** Summary: AI_A believes the young woman may be suffering from an adjustment disorder or low self-esteem due to the end of a significant relationship, rather than clinical depression. The recommended response is to reassure her that everyone makes mistakes and that her worth is not tied to occasional errors. AI_B also suggests that the woman might be experiencing an adjustment disorder. The response should acknowledge her feelings, provide support, and challenge her negative self-belief without passing sweeping judgments. AI_C thinks the woman is going through a typical grieving process after a significant life change and might benefit more from supportive feedback and resilience-building than conventional psychiatric treatment. The response should validate her feelings, support her in dealing with her self-deprecating tendency, and encourage her autonomy and decision-making ability. AI_D emphasizes the importance of an empathetic and validating response, which is more connected to patient-centered therapy. The response should acknowledge the patient's feelings and struggles while also encouraging her to see her abilities positively. AI_E hypothesizes that the woman might be struggling more with a dependent personality disorder than with depression or prolonged grief. The response should be supportive, acknowledge her feelings, and reinforce her capacity to cope and make decisions. Key Differences: The key differences in the council members' perspectives lie in their interpretation of the woman's symptoms and the underlying issues. AI_A and AI_B lean towards an adjustment disorder or low self-esteem, AI_C suggests a typical grieving process, AI_D emphasizes the importance of an empathetic response, and AI_E proposes a dependent personality disorder. Question to Clarify Disagreements: How do the council members justify their different interpretations of the woman's symptoms, and how do these interpretations influence their recommended responses?. Based on your preceding response, how would you answer the original question: [A 19-year-old woman is referred to the office by her counselor for evaluation of possible depression. The counselor describes the patient as polite, conscientious, cooperative, open to therapy, and always present and on time for her weekly sessions. The patient's relationship with her high school boyfriend ended 6 months ago. She had dated him through high school, and he had ""looked out for her as a friend"" since the 6th grade. She says, ""I feel helpless and don't know what to do without him."" She admits that she often cannot make decisions, such as whether to go to college. She says, ""I always mess things up."" She is still sad and often tearful about her ex-boyfriend, but she has not had alterations in her sleep habits, appetite, weight, or energy level. She has not had any thoughts about death. Medical history is unremarkable and she takes no medications. BMI is 24 kg/m². Vital signs are normal and physical examination discloses no abnormalities. Which of the following is the most appropriate initial response to this patient's self-deprecating description? (A) ""College is full of many great once-in-a-lifetime opportunities. You should go."" (B) ""Everyone makes mistakes. In your case, though, that doesn't mean you always mess things up."" (C) ""If you find yourself needing some guidance, call me."" (D) ""You're overreacting. You just need to give it a little more time.""]

**AI_C:** After detailed discussions among the AI council members, the young woman's condition could be categorized as a non-clinical psychological issue, possibly an adjustment disorder, low self-esteem, or dependent personality disorder. It is important to keep in mind that the absence of hallmark signs of depression, like changes in sleep, appetite, weight, energy level, or ideas of death, support this interpretation. The council has weighed and evaluated the alternatives, including the possibility of depression, adjustment disorder, dependent personality disorder, and normal grief reaction to a significant relationship loss. After thorough analysis, the woman's excessive reliance on her ex-boyfriend for making decisions, feeling of helplessness without him, and self-deprecating statements stand out as more significant indicators of her mental state. This consideration led council member AI_E to lean towards a hypothesis of a dependent personality disorder. Addressing such self-deprecating thoughts and enabling her own decision-making competencies seem crucial in this scenario. Option (B), "Everyone makes mistakes. In your case, though, that doesn't mean you always mess things up," therefore, seems to be the most clinically suitable response. This choice validates her feelings and experiences while also challenging her negative self-perceptions gently, doesn't push unsolicited advice upon her, avoids potential invalidation of her emotions, and ultimately boosts her confidence about her decision-making abilities—an aspect she seems particularly unsure of in her current state. Therefore, the council collectively believes that the answer to the question about the most suitable response is (B), "Everyone makes mistakes. In your case, though, that doesn't mean you always mess things up." This response acknowledges the patient's emotions, provides reassurance, and offers gentle corrections to her negative self-image—all in a supportive manner, without undermining her autonomy.

**AI_A:** Restating the Question: The query requires us to select the most appropriate response to a young woman who is presenting with feelings of helplessness, self-deprecation and indecisiveness following the end of a long-standing relationship. It is clear she is struggling emotionally though she doesn't meet full criteria for a depressive disorder as she has maintained stable sleep, appetite, weight, and energy levels and has no thoughts of death. Planning my response: The stated symptoms (helplessness, decisional difficulty, self-deprecation) manifest as part of a complicated emotional process. Assumptions include the possibility of Adjustment Disorder, Dependent Personality Disorder, or even simple grief reaction. Each requires a different approach but all start by validating feelings and avoiding judgmental or dismissive comments, encouraging independence and reinforcing inherent strengths. Clinical Reasoning process: The assumption here lies on understanding her symptomatology, her relationship history and the lack of typical depressive signs. Dependent Personality Disorder (DPD) is characterized by a pervasive fear of being alone and needing others to take responsibility for one's life, while Adjustment Disorder happens when a person cannot adjust or cope with a particular stressor, like a major life change. In both cases, non-angular support is key, but in DPD, reinforcing independence is critical, in Adjustment Disorder, focusing on resilience might be more beneficial. Concluding argument: While all answer options lack some ideal element, option (B) appears the most suitable. A caregiving role by the mediator (answer C) could reinforce dependent traits if present. Dismissing her feelings (answer D) or pushing her into an important decision (answer A) could increase her anxiety. So, although not perfect, reassuring her that mistakes are part of life and do not necessarily translate into total failure can challenge her cognitive distortions, which is helpful regardless of the exact diagnosis. Ultimate Response: (B) "Everyone makes mistakes. In your case, though, that doesn't mean you always mess things up."

**AI_D:** RESTATING THE QUESTION: The question involves a young woman who is seeking help for feelings of helplessness and negative self-perception following the end of a long-term relationship. Her physical health appears normal and she doesn't meet the classic criteria for clinical depression. Hence, the question wants to determine the best initial response to her concerning self-description. PLANNING: Assumptions could be that she is suffering from depression or an adjustment disorder after her break-up. Another assumption could be that she has a dependence issue, based on her inability to make decisions in her boyfriend's absence. However, the absence of physical symptoms or thoughts of death makes clinical depression less likely. Based on her self-deprecating speech and inability to make decisions, an assumption of a dependent personality could be prioritized clinically. CLINICAL REASONING: A dependent personality disorder, characterized by a chronic need for acceptance, reassurance, and support from others, would align well with her symptoms, especially her inability to make decisions. This condition often arises in young adulthood and can hinder personal development, decision-making, and interpersonal relationships. Therefore, an ideal response for her would validate her feelings but also challenge her self-deprecating thoughts and promote self-reliance. CONCLUDING: The most appropriate response should help the patient recognize her worth and strength without minimizing her feelings. Ideally, it should encourage her to independently navigate through life's challenges, build her resilience, and cope with future stressors effectively. RESPONSE: Based on the clinical reasoning, the most appropriate response would be (B) ""Everyone makes mistakes. In your case, though, that doesn't mean you always mess things up."" This response acknowledges her feelings, challenges her negative self-perception, and encourages a more balanced perspective.

**AI_B:** RESTATING THE QUESTION: The question revolves around the best initial response to a 19-year-old woman who's been referred for possible depression. She has recently gone through a break-up and has self-deprecating thoughts, though her symptoms don't fulfill criteria for major depressive disorder as there are not significant changes in her sleep, appetite, energy, or any death thoughts. PLANNING: The assumptions taken so far are that she may be dealing with an adjustment disorder, low self-esteem following her break-up, a typical grieving process or a dependent personality disorder. Generating an alternate assumption could be she's encountering normal, though emotionally stressful, reactions to a significant life change. And the assumption we should clinically prioritize, in my opinion, is this last assumption because her symptoms are not severe or long-lasting enough to fit into a clear-cut mental disorder. CLINICAL REASONING: Exploring this chosen assumption in more detail, in healthy individuals, a break-up can cause acute stress or emotional pain, but doesn't necessarily indicate a mental disorder unless symptoms persist or are severe enough to interfere with daily life significantly. Her self-deprecating thoughts and the difficulty in decision-making may be due to situational factors, perhaps aggravated by the ending of a long-term relationship where she might've relied heavily on her partner–hence feeling helpless without him. CONCLUDING: Taking these points into consideration, it seems likely our patient is experiencing a tough emotional period after her break-up, rather than a specific mental disorder. She might benefit from supportive validation and empathy to help her cope with her emotional stress and her self-deprecating thoughts. Encouraging her independent decision-making can also be beneficial as it would empower her and challenge her self-deprecating beliefs. RESPONSE: Based on this reasoning, the most appropriate initial response to this patient's self-deprecating description will be (B) "Everyone makes mistakes. In your case, though, that doesn't mean you always mess things up." This response not only validates and acknowledges her feelings, but also directly counters her negative belief system and fosters a more realistic perspective. I chose this option because I believe this statement will support her emotionally, which is what she needs the most in her current situation.

**AI_E:** (1) RESTATING THE QUESTION: The case study presents a 19-year-old woman who is exhibiting signs of emotional distress following the end of a long-standing relationship. While she is described as polite, conscientious, cooperative, and committed to therapy, she voices a sense of helplessness, lack of self-confidence, and indecisiveness, particularly regarding her future plans such as attending college. Despite her emotional turmoil, she exhibits no disruptions in sleep, appetite, weight, energy level, or suicidal ideations. The question asks for the most appropriate initial response to her self-deprecating statement, and provides four potential replies to be assessed. (2) PLANNING: Before we proceed, a few assumptions need to be identified. For instance, we assume that her counselor believes she might be suffering from depression due to her emotional state. This assumption can be challenged given that she doesn't manifest the cardinal symptoms of depression such as alterations in sleep, appetite, energy level, or thoughts about death. Another assumption is that her emotional distress is rooted in her breakup which she is yet to overcome emotionally. One assumption that we should clinically prioritize is that this young woman might not be suffering from depression, but rather from a condition related to dependency or adjustment disorder. (3) CLINICAL REASONING: Given the absence of classic symptoms of depression such as changes in sleep habits, weight fluctuations, a decreased energy level or suicidal ideations, the diagnosis of depression appears less plausible. Furthermore, the fact that her emotional turmoil followed a significant relationship breakup suggests the possibility of an adjustment disorder caused by this major life change. More importantly, her statements suggest a possible dependent personality disorder, characterized by a pervasive and excessive need to be taken care of, leading to submissive and clinging behavior and fears of separation. Her lack of decision-making ability, expression of helplessness and the weight she places on the former protective relationship hint towards such a diagnosis. (4) CONCLUDING: In light of this clinical reasoning, our initial response should aim to encourage her independence while expressing empathy and acknowledging her feelings. It should optimally steer away from implying any decisions on her behalf or general dismissive statements. (5) RESPONSE: Therefore, the most appropriate response to this patient would be option (B) ""Everyone makes mistakes. In your case, though, that doesn't mean you always mess things up."" This response implies the understanding and validation of her feelings, while also challenging her negative self-perceptions and promoting a healthier self-image. It neither imposes decisions nor invalidates her emotional experience, and facilitates further discussion of her fears and beliefs.

**Facilitator** The consensus among the AI council members is that the young woman's condition could be categorized as a non-clinical psychological issue, possibly an adjustment disorder, low self-esteem, or dependent personality disorder. The absence of hallmark signs of depression, like changes in sleep, appetite, weight, energy level, or ideas of death, supports this interpretation. The council has weighed and evaluated the alternatives, including the possibility of depression, adjustment disorder, dependent personality disorder, and normal grief reaction to a significant relationship loss. After thorough analysis, the woman's excessive reliance on her ex-boyfriend for making decisions, feeling of helplessness without him, and self-deprecating statements stand out as more significant indicators of her mental state. Addressing such self-deprecating thoughts and enabling her own decision-making competencies seem crucial in this scenario. Option (B), "Everyone makes mistakes. In your case, though, that doesn't mean you always mess things up," therefore, seems to be the most clinically suitable response. This choice validates her feelings and experiences while also challenging her negative self-perceptions gently, doesn't push unsolicited advice upon her, avoids potential invalidation of her emotions, and ultimately boosts her confidence about her decision-making abilities—an aspect she seems particularly unsure of in her current state. Therefore, the council collectively believes that the answer to the question about the most suitable response is (B), "Everyone makes mistakes. In your case, though, that doesn't mean you always mess things up." This response acknowledges the patient's emotions, provides reassurance, and offers gentle corrections to her negative self-image—all in a supportive manner, without undermining her autonomy.
